# Appropriating Shakespearean graphic novels for Malaysian classrooms to create correct gender representations

**DOI:** 10.3389/fpsyg.2022.874960

**Published:** 2022-12-07

**Authors:** Hanita Hanim Ismail, Mazlin Azizan, Radzuwan Ab Rashid, Muhammad Asif

**Affiliations:** ^1^Faculty of Education, Universiti Kebangsaan Malaysia, Bangi, Selangor, Malaysia; ^2^Academy of Language Studies, Universiti Teknologi MARA, Shah Alam, Selangor, Malaysia; ^3^Centre of English Language Studies, Faculty of Languages and Communication, Universiti Sultan Zainal Abidin, Kuala Terengganu, Malaysia; ^4^Deanship of Preparatory Year, Science and Arts College, Northern Border University, Turaif, Saudi Arabia

**Keywords:** graphic novels, gender equity, pedagogical tools, SDG goal 5, Malaysia

## Abstract

Public awareness on gender education may not be easily translated in living classrooms, which prompts new alternatives. In this study, we explored Shakespearean graphic novels in exposing Malaysian school students to gender-related issues. The two-fold research entails tracing the female presence in eight selected images from digital graphic novels that define the gender and place social expectations across the globe; and secondly, gathering teachers’ perception of these selected graphic novels. The chosen images were divided into two categories—those published as the cover pages of graphic novels and those that were contested for the Graphic Shakespeare Competition established in 2016. These stereotypical images of women are inconsistent with the objective of achieving inclusivity and correct gender representations. The data analysed based on a semi-structured interview on six ESL teachers suggest that as much as the graphic novels are seen as valuable in pedagogical contexts and in exposing the students to learning English and gender representations through literature, the material selection, and pedagogical approaches, including determining the classroom activities must be cautiously considered in terms of their cultural appropriateness to ensure students’ readiness and effective outcomes. Discussing these pertinent issues, especially in addressing gender (mis)representations relevant to education, helps pave a new route within the UN SDG Goal 5 where gender nuances and phrases ought to be carefully constructed in a new narrative that shapes global perception towards women.

## Introduction

Using authentic and literary texts for pedagogical purposes in English as Second Language (ESL) classrooms is not new; one of the challenges with this has always been material selection to fit the criteria needed for any particular teaching objectives. Material selection generally ranges beyond the use of textbooks; “they can be anything which is deliberately used to increase the learners’ knowledge and/or experience of the language” (Tomlinson, 1993, p.2), including type of texts, especially to teach literature. Yet, what remains problematic within the Malaysian context as pointed out by Mohaideen et al. (2020) is deciding between using foreign and local texts. Choosing between the two is not as easy as it seems because this leads to another question on learners’ engagement. This would explain the numerous questionable achievements of the national reading programmes created to facilitate students with reading opportunities at school levels, including English Language Readier Programme (ELRP) and Class Room Programme (CRP). Parkinson and Thomas (2000) explain that learners’ inability to engage is due to the reader’s remoteness with the text—either historically, geographically, socially, and in terms of experience.

Shakespearean texts, particularly his plays, have been generally taught to young learners elsewhere. In fact, the reading of Shakespearean plays has been reinvigorated since 2014. The efforts of making it relevant to contemporary society are observed through manga—a sub-category under graphic novels. Besides graphic novels, the first manga production was done by SelfMadeHero with its Manga Shakespeare series, which was also considered another visual format in the new publishing trend among the book industry. Publishing houses like HarperCollins and Random House were among those that took early initiatives. This was one of the several signals that indicated postmodern society’s celebration of visual culture in its daily life, including television, glossed magazines and advertising. The marriage between Shakespearean plays and manga worked well simply because the playwright intended to make his works visible, instead of readable, where the texts are brought “to life in a visual way for a new audience” (Hayley, 2010, p. 269). It was a marriage of visual poetry and textual poetry. Combining text and visuals also created a new industry for visual literacy that incorporated the ability to read, write, and create visual images where the writing aspect is taken out when it comes to the covers of graphic novels. Conner (2019) finds an evident association between reading classic literature and students’ reading attitude and achievement, especially among the boys (Lichter, 2007). At the same time, the use of graphic novels would appropriate the current need for gender equality by promoting co-education, where there are past discourses on aspects relating to academic achievement (e.g., Younger and Warrington, 2006).

There might be similar reading obstacles when it comes to choosing Shakespearean texts such as sustaining young learners’ reading engagement (e.g., Frossard, 2012; Tso, 2016) but the level of reading reception among the Malaysian school students seems to have lost its vigour, which can be explained by understanding issues regarding national text selection for reading materials. Aspects such as readers’ cultural difference and the text origins collide, thus creating continuous reactions (e.g., Gunakumaran, 2003; Ganakumaran et al., 2003; Vethamani, 2005; Isa and Mahmud, 2012; Nambiar et al., 2020). Over the decades, changes in text selection were observable when studies reported on the lack of appreciation that represented disparity in language proficiency, to begin with (e.g., Sani and Zain, 2011) to the point that rote learning can be detected among Malaysian secondary school students as a coping mechanism. Memorization is seen as the solution since Malaysian school students were required to read the Bard’s poems for their Malaysian Certification Examination (SPM)—a qualifying certificate to enter diploma and degree programmes, which affected changes in the selection of literary texts.

Unlike reading the rhetorics in argumentative writings, images are now the new form which is called visual rhetoric, and it is said to provide a pedagogical opportunity (Elkins, 2009). Mortimore-Smith (2012), for instance, applauds the effort to repackage Shakespearean plays through an appealing format among the younger readers. Its use provides an opportunity to develop higher-order interpretations. It is also essential to inculcate critical thinking skills, which thus suggests the mundane state in the book-based reading approach used in schools. With public solid reception towards visual literacy, graphic novels to help learners better appreciate literary texts bloomed since then. Besides serving entertainment, graphic novels, which are a mesh of pictures and words, aim at conveying information and producing aesthetic pleasure (McCloud, 1993). To date, the purpose of this pedagogical tool can be further maximised, especially after Inness (1999) highlights comics as bearing the potential to explore new definitions of gender, suggesting a possible route for feminism to be heard within the deconstructive effort of defining gender, particularly issues regarding female representations and roles. Even though 100 nations have signed in the Convention on the Elimination of All Forms of Discrimination against ↑Women (1979) or known as CEDAW, there are still nations that fail to exercise the promoted rights, including gender equality [Status of Ratification (n.d.)]. Malaysia, for example, has initiated ratification for its ill-status adhering to the treatise in 1995 but still at 5–9 level (Sustainable Development Goals Report, 2021). Studies of graphic novels have long certified the credibility of graphic novels as a pedagogical tool, especially in improving literacy (e.g., Carter, 2007; Fisher and Frey, 2007; Chun, 2009). It promotes learners’ autonomy in a two-fold objective, but it is also well received. While studies have shown that Malaysian students appreciate literature better through the use of graphic novels, there are also studies on learners’ difficulties to read literary texts as part of an English as a Second Language classroom experience (e.g., Abu Bakar et al., 2020) as in reviews as well as those that featured teachers’ perspectives on using graphic novels as a teaching tool (e.g., Yusof et al., 2017). Therefore, it would be interesting to explore graphic novels as a multimodal material further and examine its impacts on teachers’ pedagogical practices. It is also an opportune time to explore many potential benefits in the future, including the role of literature as enabling the move of social sustainability, specifically in vocalising gender disparity (Ismail, 2019).

This two-step research includes an analysis of eight selected images and focus group interviews as its method. These two methods were incorporated partly because more research studies are available on other uses of teaching tools, including ICT, graphic novels but none looked into the usefulness of Shakespearean graphic novels, particularly in Malaysia. The interviews served the purpose of further validating the researchers’ analysis of the eight images. For this research, these images are narrowed to analysing the cover page of these published graphic novels, as explained in Table 1. Out of eight graphic novels, two were illustrated by women—Sonia Leong and Kate Brown. This paper aims to draw an understanding on the suitability of selected images to teach on female representations, which will be explained further in the Method section. The following are its two research questions:

- What are the patterns of female representations within the selected images?
- How do the images influence teacher pedagogical practices?

**Table 1:** Scope of analysis as according to publishing houses.

	Items	Illustrators and Year
1.	Macbeth the graphic novel	Volley et al. (2008)
2.	Romeo and Juliet (Graphic Shakespeare)	Espinosa (2008)
3.	Romeo and Juliet The Graphic Novel	Volley et al. (2009)
4.	Othello Graphic Shakespeare	Allen (2008)

Since Shakespearean plays are acclaimed relatable texts across time, their values should be transferable through perceived practical pedagogical approaches. Furthermore, the use of literature has also been argued to be a practical pedagogical approach in language learning (Almeida et al., 2020).

## Literature review

### Gender representations and women issues in Malaysia

Unlike sex, gender is non-physical where its definition is socially constructed. In fact, Ramet (1996) explains that such a definition associated with gender is an encultured act where Burke (1992) associates women with specific qualities within the eighteenth-century specifics that restricted women (modesty, humility, silence, and chaste), which defines social expectations on women such as being beauty (McGarr, 2008; Specht, 2015; Ismail, 2017) and the expectation to maintain social decorum (McKeon, 1995). Gender representation when it is related to women is also associated with gender roles like cooking and child care. Others might also define expectations within different genders where the more traditional expectations of gender roles for women include their fragility when defending themselves or having to wear a dress. On the other hand, traits of masculinity are identifiable in terms of physical strength and the tendency towards violence and defensive mode, which thus substantiates Burke’s definition of sublimity for men. Men are generally expected to be brutish. To date, the image of women has been extensively researched from numerous perspectives, including their stereotypical representation in television advertisements (e.g., Kumar, 1989; Kitsa and Mudra, 2020), social media (e.g., Fardouly et al., 2015; Mills et al., 2018), and even parliamentary presence (e.g., Fokum et al., 2020), which reveals a trending pattern of recognizing women as key players in society. At Malaysian level, research on women representation is receiving a growing attention, including those on politics and public administration (e.g., Yeong, 2018; Lim et al., 2019), textbooks, and education (e.g., Mukundan and Nimehchisalem, 2008). A review on analysing women images in Shakespearean plays indicates interestingly numerous directions. While some look into the analysis of images such as those concerning roles (Kemp, 2010) and female impersonation and its connection with its political representation (Callaghan, 2002), others observe the more applied aspect of representation such as daily representation of women in staged performance (e.g., Hodgdon, 1998) and painting (e.g., Benton and Butcher, 1998).

In contrast to Lynda’s (as cited in Ahmad and Abd Ghani, 2016) assertion that Malaysia has developed numerous acts, policies, and mechanisms that promote women inclusion (including in reviewing gender equality), the nation may not have shown equally as progressive as compared to their Japanese counterparts when it comes to policy making. Up till 2021 alone, Japan has drafted more than 20 white papers and reports on issues regarding COVID-19 and its challenges to gender equality, besides work opportunities, education, and health, to name some. O’Hagan (2021) has conducted a global study on the effects on gender after school closure due to COVID-19 and one of the main goals is to safeguard gender equality in education. Among the key findings discussed are worrying gender related effects such as lack of access to digital remote learning facilities and comprehensive sexuality and health education. The National Policy on Women (1991) was the most recent policy addressing women’s welfare, including a national effort to create public awareness on women issues under the Malaysian Department of Women Development (Sixth Malaysia Plan, 1991-1995). Ahmad and Abd Ghani (2016) note the focus of the policy, which is on placing women in managerial positions. Such an initiative would benefit equal sharing between the two genders, including knowledge sharing and other aspects of life. Kelantan is ahead of the rest at the state level, revising its policy on women (Musa et al., 2018). National concerns on women empowerment and gender mainstreaming have also appeared in research (e.g., Zainuddin and Khalid, 2018; Awang and Nordin, 2019), including a progress report in the Sustainable Development Goals Voluntary National Review 2020 (First Global Workshop, 2019).

### Evaluation as part of the textbook selection and evolving issues in Malaysia

When developing international textbooks, well-developed ones are often recognised for their ability to generate an inclusive learning environment; one of the aspects needing attention is the inclusion of representations from different identities and free from stereotypes and prejudice (Fuchs et al., 2020). This includes employing an inclusive language that addresses the disparities. Within this aspect, compelling conversations can launch based on the content of well-developed textbooks (Roth and Aberson, 2006). Pingel (2009) explains that books play a role that “reflect the traditions a society has formed over decades” while allowing relatable examples and experiences that students can associate with (p.7). A textbook which is capable of engaging its users is aligned with the UNESCO (1995) Declaration and Integrated Framework of Action on Education for Peace, Human Rights, and Democracy, including eliminating negative stereotypes.

Well-selected textbooks, along with supplementary texts, are assurances for effective learning. However, such confidence yields two types of evaluation: (1) predictive and (2) retrospective evaluation (Mukundan, 2007; Mukundan et al., 2011). Despite past local studies having shown continuous scrutiny over text selection (e.g., Mohaideen et al., 2020; Nambiar et al., 2020), proper evaluation of text selection ought to be executed. While the predictive evaluation is carried out at the initial stage, the latter type of evaluation helps scrutinise the relevance of the textbook selection, looking at its strengths and weaknesses (Mukundan et al., 2011). Within the predictive evaluation, teachers can opt for the implicit or the explicit model through the use of popular checklists, e.g., Skierso (1991), Cunningsworth (1995), and Byrd (2001). Byrd (2001) highlights a need to synchronise a fit between students’ needs and the text selected. The most important two are content and presentation/format, where several questions were further itemised, including (1) Does the book look right for these students? (2) Are the illustrations and other graphical and design elements appropriate for their age and educational level? Of course, studies look into the impact of colour choices on learners’ emotions, as suggested by Pope et al. (2012). Other aspects that needed to be considered include culturally inappropriate content in imported textbooks and supplementary (Khoo and Knight, 2015).

### Pedagogical tools to teach literature for awareness in gender equality

A quick review on pedagogical tools used to create awareness reveal numerous strands formed. When Frossard (2012) suggests, reducing the level of language difficulty as a way to draw more reading interest among the young language learners, this provides an apt direction for the use of other didactic tools to create awareness and discuss gender equality such as graphic novels and manga. The use of literary texts can still serve a great function to this purpose. In fact, the issues of graphic novels as an effective teaching tool have been discussed in many studies. To begin, using graphic novels in language classrooms offer immediacy when reading. It seems that young learners struggle with maintaining focus with longer reading materials, which can be solved through the use of graphic novels (Frossard, 2012). Öz and Efecioğlu (2015), in another instance, have explored the issue of utilising graphic novels in English classrooms in Turkey where they found out that the role played by graphic novels in shaping more understanding on the subject matter has been found to be significant. They highly recommended graphic novels to be integrated into the school syllabus since these materials offer a different appeal to the students unlike the traditional teaching materials. However, even though the findings suggest that graphic novels can increase students’ motivation level to a certain extent, they warned the teachers against selecting materials that do not fit a certain criteria or cater towards the students’ demographic profile or their sociocultural influences However, using graphic novels as pedagogical tools in India was not well received. Most teachers felt their inadequacy at handling technology-based mediums, suggesting that such skill needs to be fostered first at teacher training. Such inadequacy thus reveals a generational gap at handling technology (Sinha and Malshe, 2017). Burger (2018), on the other hand, focuses on how graphic novels can be introduced to illustrate important issues on gender, sociology, or history in educational settings in order to boost students’ ability in looking at familiar materials from different perspectives while engaging their multiple and multimodal literacies.

Manga, as a sub-category under graphic novels, has long been introduced in classrooms, and it has been found to be effective as educational tools (Tamada, 2008; Murakami and Bryce, 2009; Yoshida, 2013). Okubo et al. (2019) studied how manga has been adopted as an authentic material and an effective teaching method in teaching reading by looking at the semantic content and they found positive effects on the comprehension process dealt by elementary school students. There are also studies on manga with the focus on gender representations found in the narratives (Johnson-Woods, 2010; Danziger-Russell, 2013), but without the educational purposes as their focus. However, even though lacking, there are studies on manga which not only look at how it can be an educational tool, but which may also help us to better understand gender representations and their effects on society. Unser-Schutz (2011) specifies two ways that manga can be approached as an educational tool: content-based or language-oriented approaches, both with their own advantages and should be chosen according to the focus of the teaching and learning process determined by the teachers. She further explains the two types of manga for this purpose: manga for education and manga that are chosen for the purpose of teaching a particular topic, with the second being authentic texts, aiming to have people study Japanese culture and society through the narratives. Focusing on the linguistic features in manga, she found that gendered language that are featured in manga are written almost too unrealistically and with stereotypes compared to normal usage in real life especially for female characters and therefore might be giving a false impression on how females actually use language.

## Materials and methods

Despite the abundance of illustrations available in the market, which may even vary in language, region and publishing houses which feature gender representation (including those that are listed by the Notable Books for Children), only eight images were used in this qualitative research which were drawn from two sources—(1) images from published graphic novels and (2) those submitted for a Shakespeare-associated competition. Since the research was set out to test whether Shakespearean image-based texts would serve as invaluable pedagogical tools to learn English, the selection focused on the following criteria of selection:

- *Type of text*—based on any of Shakespearean plays
- *Scope*—incorporation of both images and words
- *Presentation*—those that featured images, instead of entirely texts as a means to attract readership, including graphic novels, manga, and comics.

The following sub-section will discuss further on the selection of images. Based on the selected images, a focus group interview was employed to extract teachers’ feedback on the impacts of text selection on their pedagogical practice.

### Research instruments

#### Selected graphic novels

For this research, these images are narrowed down to analysing the cover page of these published graphic novels, as explained in Table 1. We looked at seven out of 39 Shakespeare-related graphic novels, and two were illustrated by women—Sonia Leong and Kate Brown. Image selection was purposive to fulfil the expectations of the research questions based on two criteria: (1) graphic novels that feature the representation of women, and (2) the texts that were based on Shakespearean original works.

To take up the second direction in examining women representations based on Shakespearean texts, our search was also focused on Graphic Shakespeare Competition, an internationally recognized contest that pooled different talents in selecting texts and images to best represent certain themes or themes concerns. Images were acquired mainly from two websites (Table 2)—(a) https://elsinoregraphicshakespeare.tumblr.com/; and (b) https://www.facebook.com/groups/1015421885177194/?fref=mentions. The Graphic Shakespeare Competition is deemed to be an established competition, which has run since 2018 and recognised by the Asian Shakespearean Association,^1^ thus justifying its selection. These two websites provide sources of submitted images meant to be contested.

**Table 2:** List of competed graphics at the Graphic Shakespeare Competition.

	Items	Illustrators
1.	Macbeth, Act 1, Scene 5	Briggs
2.	Macbeth, Act 2 Scene 1	Ari Jogaiswara Adipurwawidjana
3.	The Three Witches in Macbeth, the Red King	Ascari and D’armini
4.	The Three Witches	Lynch and Lynch

Besides these images, we have also featured an independent work by Stefano Ascari and Simone Dármini, which was submitted *via* a similar Tumblr that contributed towards the analysis of the Three Witches. The selection of these images was based on the attempt to trace women representations in graphic novels and those related. The piece was scrutinised based on the inclusion of women as images in graphic novels and graphic images.

#### Focus group interviews

To answer the second research question, semi-structured online focus group interviews were conducted to gather teachers’ feedback based on photo-elicitation, mainly to discuss its suitability of representing gender. A photo-elicitation uses images “to invoke comments, memory and discussion in the course of a semi-structured interview” (Banks, 2018, p. 76). Initially, calls for participation were made across different WhatsApp groups, which were thought to enable a diverse understanding about teachers’ perception of the graphic novels. Yet, only a few responded, which allowed the administration of this method. Some of the respondents were connected *via* snowball sample selection; they were introduced to the researchers *via* earlier identified respondents. In the end, a total of six participants were selected, comprising two types of English teachers (Group 1: *F* = 3; Group 2: F = 3; M = 1). The first group comprises three teachers (*N* = 3 F) with more than 18 years of teaching experience from urban to semi-rural areas. Meanwhile, the second group comprises three teachers (*N* = 1 M; 2 F) with 10 years of teaching experience residing in Sarawak and Perak. The latter group is mainly teachers within option; having selected to focus on teaching English as Second Language (TESL) during their teacher training, while the former group comprises teachers “out-of-option” where they are not graduates of TESL. Despite their different training backgrounds, both groups of teachers showed equal level of awareness of students’ inability to cope with foreign reading texts. The second group is mainly young teachers (ranging from 5 to 10 years of teaching experience). A balanced selection between within and out-of-option teaching background would provide a richer outlook on aspects such as reader reception, text suitability, etc. Since most of them are from the sub-urban and remote areas, their first-hand experience with students’ struggle with literature is vivid and reliable. These two-layer focus groups would allow the saturation of images (Banks, 2018). An interview protocol was designed, which includes the following questions:

1. What do you think about using these nine images to discuss women’s representation as part of an English classroom?
2. How would you design your teaching approach using these images?
3. Would Shakespearean graphic novels be accepted by Malaysian primary and secondary school students? Why? Why not?
4. What are the strengths, weaknesses, and suggestions on using Shakespearean graphic novels?

A photo-elicitation would enable a discussion over photographic multivocality and its impact on human social relations (Banks, 2018).

### Thematic analysis

Thematic Analysis (TA) of Braun and Clarke (2006) was used twice in this research – first, analysing the selection of the graphic novels, and secondly, analysing the data from the focus group interviews. This study maintained Braun and Clarke’s proposed six phases to examine the selected graphic novels, as shown in Table 3.

**Table 3:** Six phases of thematic analysis (TA) of Braun and Clarke (2006).

Steps	Phases
1	Familiarization
2	Developing initials
3	Searching for themes
4	Thematic review
5	Thematic definition
6	Write-up

To answer the first research question, the researchers identified and scrutinised available graphic novels and graphic images that were available on the Internet mainly on two criteria: (1) texts which are based on Shakespearean plays and (2) its inclusion of women figures. Then, we organised and analysed the initial understanding of the selected graphic novels and images in Phase 1 to avoid biases. Once a set of images chosen was formed, familiarising with the images started where notes were taken to shape thematic directions. These notes then allowed Phase 2 to happen. In order to achieve validity and reliability, an investigator triangulation was employed (Denzin, 2009) where each researcher was ready to form an initial understanding of the images, without the interference of another’s opinion over the images. This understanding would have included their personalities and background as Malaysians. This also enabled convergence (Matthison, 1988). Thematic patterns were then identified across the images, allowing meaning and similarities to be drawn upon, ensuring reliability (Braun and Clarke, 2014). What followed was the search for themes, where reconsidering took place to achieve some possible ones. Unlike tracing lines that reiterate those themes, we carefully looked at suggestions within the images that supported the two themes, which are identifying patterns of women representations, and using these representations as teaching materials. Within the first theme, evident sub-themes were observed (1) evil women, and (2) women in love, while within the second theme, looked into possible teaching suggestions in relation to the use of these images in Malaysian school context. In the fourth phase, we re-examined the themes to measure our claims’ realities. Here is where we thought hard on aspects that concern women (e.g., sense of attraction, etc.). In the fifth phase, we derived definitions of both themes and proceeded to the final step which was to write a report that synergised between specific evidence in the images (as provided in phase 1), analysis (as provided in Phase 3 and 4), and contextualising the analysis to build an argument (in Phase 5).

To answer the second research question, data from the semi-structured focus group interviews were transcribed and analysed using Atlas.Ti Cloud. Using Atlas.Ti Cloud was enabled through initial developed coding that provided quick detection of similarity or difference of opinion within the two groups. Since the trial pack offered by Atlas.Ti Cloud features colour highlights, this allowed easier identification of potential codes and its analysis. This provided clearer identification of four themes: material selection, reader reception, representations of women, and how to teach. With each occurrence of agreement, disagreement, or extensions of opinions, analysing the transcribed interview sustained the process of finalising the themes. It was also noted that differences of experiences mainly occurred due to the participants’ geographical setting or length of teaching experience. These transcripts were then read again to saturate further aspects that help improve the themes, shaping the three pieces as discussed in the Results and Discussion section. In order to achieve reliability of the analysis, the analysis was done independently, which revealed to an extent, a richer and holistic observation of the subject discussed.

## Findings and discussion

This section is divided into two parts: first, to answer the first two research questions and second, to address better compelling functioning graphic novels as a pedagogical tool that help elevate the positioning of women in societies. Findings revealed the two main scopes that in review provided an understanding of the images, which thus provided the basis for further analysis and discussion.

### Patterns of women representations

#### Evil women

Stirring away from the domains of socially expected female characters, the first theme that caught our attention is evil women. As observed in Shakespearean plays, identifiable women include Lady Macbeth, Tamora from Titus Andronicus and the three witches. Both Lady Macbeth and Tamora are depicted as women who have far-vision, knowing what they want. Shakespeare might not have been familiar with writings Burke (1992), but the former was from a century that failed to acknowledge women’s importance. The English Renaissance was a patriarchal society; only a few outstanding women called out respect, including Queen Elizabeth 1. Women remained as second-class citizens before late modernism and postmodernism. As cited in Fleming, (2016), Clement Shorter describes women during the Renaissance as the other sex where they were perceived as a subject that deserves social dejection. They were denied most of their rights, including the right to have property and freedom of speech (Ismail, 2017). Thus, violating these social expectations towards women would explain how women quickly are associated with evil. When women lose their essence of goodness, they appear to be creatures of disgust or terror, which befalls the men’s realm.

Lady Macbeth is the first image of an evil woman in Shakespearean plays. Her combined image as one who is capable of treacherous schemes to triumph Macbeth’s campaign at becoming King and uncontrolled jealousy at supposing a scandal between her husband and Lady Duncan appropriately anoint the common expectation of a good woman; one who is supposed to be modest and chaste. Lady Macbeth is one of Shakespeare’s several evil characters. When she purchases the poison from the succumbing Doctor of Physick, it is motivated by ambition. Yet, is she truly as evil as she is associated with? Her sleep-walking episode reveals a long-standing counter-argument. Despite her wishes for Macbeth to become King, she subconsciously purges her silence in doing evil, which is later followed by her suicide (Kocher, 1954). It was a guilty conscience. The covers from the graphic novels are divided in their representations. On the one hand, Volley, Devlin, and Campbell depict Lady Macbeth from an angle that suggests conniving schemes and the ability to execute such plans (Volley et al., 2008) where her sneers are firmly embedded within her eyes. The illustrators’ choice to focus on the eyes provides intensity to the intended emotion, which readers can easily relate with. This steers far from the conventional depiction of women with beauty (McGarr, 2008; Specht, 2015; Ismail, 2017).

On the other hand, Briggs illustrated the character as one with innocence (Briggs, 2020a,b). There is a noticeable difference between both sets of eyes. While the image displayed by Volley, Devlin, and Campbell (Volley et al., 2008) reiterates general presumption about Lady Macbeth as an active agent of Evil, Briggs’s depiction of innocence (Briggs, 2020a,b) helps understand Lady Macbeth as a supportive wife to her ambitious husband. It is also interesting to include Adipurwawidjana’s illustration of Lady Macbeth that features Indonesian culture (Adipurwawidjana, 2018). Without the intensity of her eyes, the illustrator crafts the character to embody his conceptual understanding of the character based on his context.

Of course, the more familiar female image is the set of three witches, and in an earlier illustration of the three characters, Lynch and Lynch craft the three witches using a humorous slant that suited the current readers. Reiterating Zanettin (2010), featuring funny looking witches, helps deliver the intended humour in the plot twist. Unlike the more common adaptation of the three witches as those that are not beautiful and eerie in nature, crooked teeth flashing older women convey the humour, which probably serves as a social criticism towards the present society and their too-frequent use of social media and selfies. This was, however, approached differently by Ascari and Dármini, who illustrated the three witches as part of projecting a Gothic image in representing an overall impression about Macbeth. Interpretation of Macbeth of Ascari and D’armini (2020) as the Red King (Ascari and D’armini, 2020), the three witches were reinterpreted differently from the more familiar conventions.

The three witches have been coined differently in separate times—Polanski (1971), Wright (2006), and Goold (2010), as seen in Lynch (2016a,b). Yet, Ascari and D’armini (2020) suggest gothic imagery of the three witches (Macbeth).

#### Women in love

Among the few female characters that portray womanly emotion are Juliet and Desdemona. With over 62,500 in the Google Scholar alone, Juliet as a character has been researched from many angles. An interesting revelation is that most of these results included those that also paid attention to Romeo; Juliet is rarely studied on her own, indicating that she is co-dependent of Romeo for a research-worthy topic. Some of the more exciting research directions taken at understanding Juliet is her comparison to other female characters like Desdemona and Ophelia (e.g., Chong, 2012; Fleming, 2016) or her role as a daughter (Hamilton, 1943). There are also studies on her suicidal act, mainly focusing on the metaphor of the dagger (e.g., Duncan-Jones, 1998). This lack of focus on Juliet as a research topic somehow helps explain her standing position. She is either illustrated to highlight her womanly figure or accentuate her most sensuous physical features‑her backside and bosom (Espinosa, 2008; Volley et al., 2009). Apart from that, her gaze is always locked on her male companion, suggesting her co-dependence on his existence.

Similarly, Desdemona is also illustrated without revealing her facial expression (Briggs, 2020b). This can provide a presumption of the lack of identity. She remains anonymous unless she is associated with Othello the Moor.

The lack of acknowledgement towards female presence as characters says length about the continuous assessment on the importance of women in society and their visibility. This is one form of visual discrimination against women, albeit her fictional presence. A blog run by Oxford University Press (OUPblog) acknowledges the disparity between male and female representations in Shakespeare’s plays (2015). Their voices and roles are lesser than men, notably in most of the spaces. This further confirms description of women’s position of Kemp (2010) during the Shakespearean society where they could only elevate their status within the peripheral column by getting married. Despite Othello as a play featuring domineering husbands (either directly or indirectly), graphic novels have the potential of deconstructing messages within the images to educate the public, which in this research, suggests empowering women.

### Using Shakespearean graphic novels as pedagogical practices

The focus group interviews revealed two central perceptions observed upon understanding the impact of using the selected graphic novels as pedag ogical tools for correct gender representations.

#### Using representations of women as teaching materials

Volley et al. (2008) receives differing opinions from primary and secondary teachers when discussing the represented gender. One of the participants observed an uneven distribution of attention where “more focus” is given to the male character,” suggesting the female character is overshadowed. IHe added that the female character is always perceived as a “sidekick,” which is a prolonged socially underprivileged status. However, another participant highlighted the character’s eyes seen in Volley et al. (2008) (which is also observed in Briggs, 2020a) as synonymous to women, thus suggesting their strength and power, despite her loose tresses that suggest frailty within the gender. Her exact words of the eyes are women’s “powerful representations.” The eyes seem also to show women’s drawing features (Briggs, 2020b). Another participant, who is also a secondary teacher, echoed the strength within the eyes in Volley et al. (2008)—the image does not give the idea that she is “that fragile”; “she is, in fact, someone who has ‘something like power’.” Yet, this is a flawed opinion. Unlike what is suggested by Fuchs et al. (2020), some of the images in the graphic novels do not tell appropriate representations of gender, thus failing to offer an inclusive learning environment. By perceiving women as sidekick characters, young learners would pick up wrong signals of the gender’s inclusive role in society.

Another remark is given on Lynch (2016a,b) commenting on the deliberate yet humorous illustrations of the three witches, which deconstruct the impression about women in general. A participant questioned the illustrator’s choice of presenting women with crooked noses and ill-formed physical features that might invite Malaysian school pupils to joke about it, instead of continuing the common practice of admiring women’s beauty and elegance through the image association of women and their physical presence. In fact, what is presented is three hideous looking women who are exaggerated in their ugliness. These three ugly witches can be perceived in two folds: one, they are accessories that prompt Macbeth’s last acts of murders, and second, they deconstruct the conventional expectation towards women since beautiful women are “conventionally” expected (Specht, 2015). Instead, concepts such as ugliness and evil are interchangeable. This is exactly the direction taken up when postcolonial critics observed the representation of Othello as the Other; albeit the Blackamoor’s administrative success, he remains as a “ram”—a common illustration of Evil, since Satan is depicted to be featured as an animal with two horns. Such a construction demoralises the character’s credibility, questioning his intention at manning the post given.

On another level, Lynch (2016a,b) offers a refreshing insight towards women where the witches provide comic relief as a way to engage young learners. As was remarked, students might find it humorous; the participant projected students’ level of engagement, even if the potential of making fun of the characters. Comments like, “Eh, so funny!” might surface, for example. Upon initiating an engagement in terms of presentation (Byrd, 2001), this will cause learners to explore the content. Here is an example of a transformation in women narrative that gives an advantage to marginalised and outcasted women, those who do not fit the conventional idea of women. However, the fact that what we have here are these two impressions of women that are the extreme opposite of each other may indicate the inaccuracy in women representations that supposedly should make up the cases of real women. The idea of saying that if a woman is not attractive, she has to be ugly or evil is somewhat disturbing and this, therefore, might lead towards preparing the young minds to think that this is normal and may have the grooming effects of continued expectations, especially in accepting the basis of patriarchal society.

However, it is interesting to point out an observation commented by one of the participants regarding Espinosa (2008), which promotes equality between the genders. In the image, both Romeo and Juliet are illustrated to stand, facing each other with the rest of the world against them. The Capulets and the Montagues are arch-rivals, which reject the union. Yet, the two lovers’ locked gaze signals that they are united in an instance; that their attempt to survive the odds is an equal effort. While discussions on love and happy endings bring tingles to the heart, the idea might overwhelm budding readers. Such an analysis might engage deeper conversations, which are not suitable for primary and secondary school students to relate with (Pingel, 2009). However, there is a possibility of a simplistic recognition that this might suggest something further than love and happy endings, leaving more negative effects than good ones to the students.

#### Potential issues in using the selected graphic novels

In general, only four figures (Allen, 2008; Lynch, 2016a,b; Briggs, 2020a,b) were observed to have the potential of becoming ESL pedagogical materials that educate correct gender representations in a Malaysian context. The remaining five were labelled as non-appropriate and “unworkable” for Malaysian primary and secondary students. Some of the reasons mentioned include cultural appropriateness and students’ appeal. For instance, Allen (2008), Volley et al. (2009), and Ascari and D’armini (2020) might steer parents’ concern over exposing minors to unsuitable contents (either psychological or intimacy), including dark illustrations such as those seen in Adipurwawidjana (2018) and Ascari and D’armini (2020).

Briggs (2020a,b) were notable for their amiable representations of women at one level. A participant compared between Volley et al. (2008) and Briggs (2020a,b), commenting that the lady illustrated in Briggs (2020a,b) to be “nicer” than the former. Unlike Volley et al. (2008), the latter suggested a different idea of representation on womanliness. Both images offer the richness of interpretation seemingly because “a woman has many characters” where “some women are like the first picture,” unlike the one suggested in Briggs (2020a,b). On another note, the participants raised concern over the impacts of using the selected images (e.g., parental queries, early exposure to inappropriate influences) to teach gender awareness and representations in ESL classrooms. They observed that Allen (2008) and Volley et al. (2009), in particular, expose early conceptions of intimacy which are not suitable for Malaysian primary and secondary school students. Both figures portray images that are not often welcomed warmly among the more conventional Malaysian societies, especially those involving the practice of public display of intimacy. Kissing, hugging, and innuendoes that suggest anything within those aspects are likely to cause raised brows among mostly conservative parents. Because of that, participant 1 pointed out that parents are likely to ask teachers’ purpose in material selections. Another exclaimed, adding a raised alarm over parents’ worries, simply because it can cause “cultural shock.” Malaysians, in general, are predominantly conventional in their views regarding pre-marriage relationships, especially if it concerns their young adult children in schools, thus explaining possible retention against progressive thinking and life practises. What is interesting about these observations is that the assumed worries of the parents may have stemmed chiefly from cultural and religious beliefs rather than the effects of them being raised in a patriarchal society. The Malay society, for example, frowns upon any display of public affection, which indicates, to a certain extent, the intention of preserving a particular traditional outlook in life. All these, to an extent, contradict Shanahan et al. (2019), who suggest that visual women representation in social media glamorise sexual impressions towards the gender. This conventional defence against the evasive social media is further reiterated for the need for selecting appropriate textbooks and supplementary texts that culturally suit the Malaysian learners in ESL classrooms (Khoo and Knight, 2015). Suppose the selections of graphic novels that are first intended to expose the students to positive gender representations may invite more harm than good. In that case, this will defeat the purpose of it being introduced in ESL classrooms in the first place, since the main goal should be to teach the students English.

Another feedback is also observed in terms of image presentation. It was highlighted that primary school students are more likely to react positively to vibrant images that encourage the development of good mental health. Unlike Volley et al. (2008), Adipurwawidjana (2018), and Ascari and D’armini (2020), participants from the primary schools pointed out the dullness of these least appealing images and are most likely to trigger depression among Malaysian primary pupils, which arguably may suggest the unreadiness of students in appreciating the values behind the materials. Meanwhile, some of the figures offer deep and dark messages that might cause potential depressive messages. A participant commented on the unsuitable theme observed in Adipurwawidjana (2018) and Ascari and D’armini (2020). While the darker motives employed in these figures might work among the more vibrant and well-exposed readers (maybe those who are more mature), a womanly figure donning a black attire might not engage the young learners, despite the cultural familiarity that might fit the Indonesian society. Therefore, the relational qualities of graphic novels should also be paid attention to. This echoes an earlier address on the choice of colour to suit the young learners in the promotion of reading engagement, reiterating emphasis of Byrd (2001) on synchronising students’ needs with the text. Yet, a secondary teacher expressed a perfect representation of Lady Macbeth—the person who is capable of devising Macbeth’s evil doings through the suggestion of her looks and stare, having “something on her mind; plotting.” This has come to conclude the importance of material selection which has to be based on level appropriateness and the maturity of the students in being able to accept and appreciate the graphic novels accordingly so that they will be beneficial both in educating the students on correct gender representations and also elements of the English Language.

#### Suggested teaching activities: Blessings and drawbacks

Interestingly, the other four potential ESL pedagogical materials hold weight due to the possibility of conducting activities that can accelerate reader engagement and prepare for students’ readiness to immerse into a different cultural reading. Some of the suggested teaching activities include the use of collaborative learning and dramatisation. One of the options suggested by the participants includes using these graphic novels to initiate collaborative learning. The study’s direction on collaborative learning is quickly gaining attention; its rigour has stretched to mobile technology-supported activities (Fu and Hwang, 2018). A participant suggested that providing a context for Briggs (2020a,b) would enable a practical discussion in collaborative learning. Students would be able to discuss their differing perspectives of Lady Macbeth’s eyes, for instance, leading towards bigger concepts of motives at masterminding over an evil scheme. Teachers may scaffold such discussions by prompting necessary questions that can lead to students’ greater interrogation over the character Lady Macbeth as an evil person. This may encourage students to adopt more discursive strategies in their understanding of the graphic novels. This is simply because Brigs’s portrayal of evil through Lady Macbeth might contradict between students in Briggs (2020a,b).

Besides that, one participant pointed out that the use of dramatisation and role-playing can also be used upon using the potential graphic novels, which mainly reiterates Vygotsky’s Social Interactionism in classroom learning. It would provide opportunities towards a more collaborative and participatory learning that does not only mould towards learners’ development of self-esteem but also learner autonomy. However, there is a need to prepare well towards administering dramatisation in a classroom such as doing some research into understanding the characters, where students can carry out inquiries in order to formulate a particular understanding. Based on Lynch (2016a,b) (The Three Witches), for instance, students can find out about the witches’ use of magic and the extent of its impact on Macbeth’s wrongdoings. In order to ascend the throne, Macbeth has to eliminate any possible hindrance.

Some participants reiterated that teachers need to prepare the students first by creating awareness on specific topics that might touch on different cultural exposures and appreciations before introducing the activities related to the graphic novels, which reiterates Mohaideen et al. (2020) urge for the use of local materials. Of course, this is exactly what Tomlinson (1993) describes as readiness that students need to be equipped with any form of successful interaction which can take place while experiencing an impactful material. Nevertheless, not only the careful considerations on the selection of the materials are essential, the teachers’ crucial role and pedagogical approaches in being able to plan appropriate activities to be carried out effectively in their lessons involving graphic novels should also be given great emphasis. This can lead to a possibility of using drama as a way to promote awareness on gender representation where students can co-create images that would allow correct representations of gender representation through online administered competition, similar to the one employed in Graphic Shakespeare Competition. Preparing Malaysian local students towards a more immersive reading experience through the use of literature, particularly graphic novels may raise a need for an introduction that provides early exposure to a different culture, other than those experienced by these Malaysian students. Not all Malaysian students would be able to grasp an understanding about neither magic nor its practitioners. For example, there would be different perceptions towards the impression on the magical world that would include witches and wizardry. There is a clash of understanding and connotation with regards to magic since the majority of Malaysian students are conservative in their acceptance of foreign materials.

However, due to the participants’ limited feedback with regards to the suggested activities, an elaborate discussion was disengaged as due to their teaching experience and concerns as practising teachers. One particular participant raised an issue of unfamiliarity about the portrayal of the character where she said, “*Uhh, they will say the witches look funny lah. They might not know these are witches. Like a bunch of dudes in dresses*.” Such a comment is understandable since there is a disparity of understanding when it comes to images about ugliness and women. As discussed in the section “Evil women,” Malaysian school students might misconceive ugliness, failing to identify witches to begin with.

Furthermore, it was observed that the participants or teachers involved in the focus group interviews did not provide too many suggestions on the teaching activities mainly due to them being more distracted by various concerns, especially regarding the students’ ability to accept and learn from the graphic novels and the suitability of the materials themselves, which seem to carry more importance. This is illustrated by another participant from the focus group interviews: “*These are not suitable for secondary school students. So maybe, university students yea can but secondary school they are just, tambah-tambah my place kan, kampung nanti kan, they’ll be like ‘Apa ni?’*” (*especially my place is in the rural area, so they’ll be like ‘What’s this?*’). This kind of concern should not be taken lightly since it arguably is a sign of a bigger issue when it comes to introducing new materials in Malaysian classrooms; e.g., failure in executions of new teaching materials in the past due to unmatched cultural expectations or bureaucratic red tapes. The concerns related to culture and suitability of materials might be from the teachers’ close observations over the years on students’ and societal expectations and reactions to something similar to the introduction of graphic novels. The hesitations might stem from the apprehensiveness of the Malaysian society as a whole towards the state of being led or drawn into the mould of the Western ideologies and thinking. As much as this is having prominent effects in safeguarding their identity, ideology, values and faith of Malaysians, which are understandably very important to them, this leads the teachers to become more sceptical to the issue brought forward by this study and this situation arguably might hinder Malaysia from venturing into something new. However, there are also signs of openness, which can carry promising and effective outcomes to efforts like this one if amiable conditions can be met.

Therefore, introducing graphic novels in Malaysian classrooms needs more cautious planning and creative approach. One possible way this can be achieved, which was not specifically mentioned by the participants, might be through online platforms that are deemed suitable for student consumption in conducting both class activities and online discussions. This can allow a safe online or digital space for the students, while tapping into their interests and abilities to process the information for language learning purposes. Discussion on gender representations found in the graphic novels can be one of the activities where students can compare each others’ reactions and know where they should position their thoughts and ideas to shape more knowledge and acceptance towards this issue. Teachers can play an active role in facilitating the learning process by pointing out relevant points or questions to be discussed on gender representations, controlling how the construction of knowledge should take place and ensuring proper understanding and positive takeaway messages on gender.

## Conclusion

This study highlights the issue of women representations in Shakespearean graphic novels and how they are mainly depicted as evil women and women in love. Still, most importantly, it also tries to elevate the role of women and empower them through the use of graphic novels as a pedagogical tool in ESL classrooms. A number of studies are now looking into gender (mis)representations found in the narratives of graphic materials and have discovered both disturbing and hopeful observations when it comes to understanding gender or gender roles in society (e.g., Allison, 2014; Nayar, 2017), which findings and concerns are also shared by this study. By identifying and revealing what can be regarded as visual misrepresentations of or discrimination against women, it seeks to create awareness on these pertinent issues on both teachers and students in Malaysian contexts. In review on how graphic novels should be utilised in teaching: as the medium of change to correct the false perceptions of gender in Malaysian society, it will be most fruitful to approach this through the eyes of the teachers and the young generations. Therefore, it is highly recommended that more new approaches in achieving similar aspirations of emphasising the importance of women in the society can be introduced by utilising various sources of literary materials (either printed or digital) and other researchers should be further encouraged to adopt and explore in initiating new and innovative directions, objectives, and methods in the future, be it in education or other fields. While graphic novels may steer newfound interest for reading through the use of images, feedback from selected teachers indicated concerns over material selection. Some of the highlighted worries are inappropriate cultural content and the layout of the materials that might not be suitable, especially for primary school pupils. It is also feared that it may backfire and lead to adverse effects rather than creating inclusivity and empowerment by introducing them in ESL classrooms. While it has been found that graphic novels can significantly help students to learn, especially in ESL classrooms (Öz and Efecioğlu, 2015; Abu Bakar et al., 2020) both studies also warned that inappropriate selections of the materials can be an issue. In line with this, the teachers suggested that selecting graphic novels that are more relatable and appealing enough to the students can lead to their readiness and positive appreciation towards the chosen materials and the topics raised on gender (mis)representations.

These findings revealed possible directions for future research, especially in the Malaysian context where material selection remains crucial, particularly in a society that perseveres towards maintaining its traditional life practice. Since the focus of this study is set mostly on the materials and limited to a small number of teachers, future researchers interested in this topic might want to visit this issue from the perspectives of students, parents, or even illustrators on top of a bigger number of teachers, which may provide an insightful understanding of gender representations through graphic novels. It is also recommended for future researchers to work hand in hand with the officers, as well as the decision and policy makers, especially those at the Ministry of Education, in determining the path of our education system in relevance to the issues in question, which is not addressed in this study. Rigorous research studies should also delve into studying criteria needed when choosing international texts, especially graphic novels that are impactful at building future Malaysians through appropriate content aligned with the society’s values and traditions.

## Data availability statement

The raw data supporting the conclusions of this article will be made available by the authors, without undue reservation.

## Ethics statement

Ethical review and approval was not required for the study on human participants in accordance with the local legislation and institutional requirements. Written informed consent for participation was not required for this study in accordance with the national legislation and the institutional requirements.

## Author contributions

HI designs the study and drafts the paper. MaA mentors the study design and data analysis and refines the manuscript. RR and MuA help in data interpretation and editing the manuscript. All authors contributed to the article and approved the submitted version.

## Conflict of interest

The authors declare that the research was conducted in the absence of any commercial or financial relationships that could be construed as a potential conflict of interest.

The reviewer HN declared a shared affiliation with the author HI to the handling editor at the time of review.

## Publisher’s note

All claims expressed in this article are solely those of the authors and do not necessarily represent those of their affiliated organizations, or those of the publisher, the editors and the reviewers. Any product that may be evaluated in this article, or claim that may be made by its manufacturer, is not guaranteed or endorsed by the publisher.

## Footnotes

^1^ http://asianshakespeare.org/resources/

## References

[ref1] Abu Bakar A. Y. Ahmad A. Dayang N. Yunus M. M. (2020). Students’ acceptance to using graphic novels in learning literature (L2): a Malaysian case study. Soc. Sci. Human. Educ. J. 1, 43–51. doi: 10.25273/she.v1i2.6626

[ref950] Adipurwawidjana A. J. (2018). Act 2, Scene 1. Graphic Shakespeare Competition. [Image]. Facebook. 15 March 2018. Available at: https://www.facebook.com/photo/?fbid=10154630753507609&set=pcb.1728821827170526 (Accessed November 30, 2022).

[ref2] Ahmad N. A. Abd Ghani M. (2016). Dasar Wanita Negara: Daripada Polisi kepada Pelaksanaan. J. Pembang. Sos. Policy Profession. Dev. 69–86. doi: 10.32890/jps.19.2016.11533

[ref95] Allen C. (2008). Graphic Shakespeare Othello. Adapted from Shakespeare by Vincent Goodwin. Cover page. Minnesota: Magic Wagon. Available at: https://www.amazon.com/Othello-Graphic-Shakespeare-William/dp/160270192X (Accessed November 30, 2022).

[ref3] Allison M. C. (2014). (not) lost in the margins: gender and identity in graphic texts. Mosaic 47, 73–97. doi: 10.1353/mos.2014.0042

[ref550] Almeida A. B. Bavendiek U. Biasini R. (2020). “Introduction,” in Literature in Language Learning: New Approaches.

[ref93] Ascari S. D’armini S. (2020). Three Weird Sisters. [Image]. Twitter. Available at: https://twitter.com/steasc/status/1120653236921303040 (Accessed November 30, 2022).

[ref5] Awang N. Nordin N. (2019). “Women empowerment for sustainable development through the implementation of flexible working hours” in *Proceedings of the Langkawi International Multidisciplinary Academic Conference (LIMAC 2019)*.

[ref6] Banks M. (2018). Using Visual Data in Qualitative Research. Los Angeles: Sage.

[ref7] Benton M. Butcher S. (1998). Painting Shakespeare. Journal of aesthetic. Education 32, 53–66. doi: 10.2307/3333305

[ref8] Braun V. Clarke V. (2006). Using thematic analysis in psychology. Qual. Res. Psychol. 3, 77–101. doi: 10.1191/1478088706qp063oa

[ref9] Braun V. Clarke V. (2014). Thematic analysis. J. Posit. Psychol. 12, 297–298.

[ref910] Briggs K. (2020a). Lady Macbeth (Macbeth, Act 1, Scene 5) [Image]. Facebook. 17 August 2020, Available at: https://www.facebook.com/photo/?fbid=10218135133834698&set=pcb.3327237820662244 (Accessed November 30, 2022).

[ref911] Briggs K. (2020b). Lady Macbeth (Macbeth, Act 1, Scene 5) [Image]. Facebook. 17 August 2020. Available at: https://www.facebook.com/photo/?fbid=10218135134714720&set=pcb.3327237820662244 (Accessed November 30, 2022).

[ref91] Burger A. (2018). Teaching Graphic Novels in the English Classroom: Pedagogical Possibilities of Multimodal Literacy Engagement. Cham, Switzerland: Palgrave.

[ref10] Burke E. (1992). A Philosophical Enquiry Into the Origin of Our Ideas of the Sublime and Beautiful. ed. Phillips A. (Oxford & New York: Oxford U.P. (Original work published 1759)).

[ref11] Byrd (2001). “Textbooks: evaluation for selection and analysis for implementation,” in Teaching English as a Second or Foreign Language. 3rd *Edn*. ed. Celce-Murcia M. (Boston, MA: Heinle Cengage Learning).

[ref12] Callaghan D. (2002). Shakespeare Without Women: Representing Gender and Race on the Renaissance stage. London & New York: Routledge.

[ref82] Carter J. B. (2007). Transforming English with Graphic Novels: Moving towards our “Optimus Prime”. The English Journal 97, 49–53.

[ref96] Chong P. Y. C. (2012). Shakespearean Character Fictions: Contemporary Re-presentations of Ophelia, Desdemona and Juliet. Unpublished Master Thesis National University of Singpore.

[ref13] Chun C. W. (2009). Critical literacies and graphic novels for English-language learners: teaching Maus. J. Adolesc. Adult Lit. 53, 144–153. doi: 10.1598/JAAL.53.2.5

[ref14] Conner K.R. (2019). Building bridges: Connecting to the classics with young adult literature. PhD Dissertation. Gardner-Webb University.

[ref15] Cunningsworth A. (1995). Choosing Your Coursebook. Oxford: Macmillan Heinemann.

[ref16] Danziger-Russell J. (2013). Girls and Their Comics: Finding a Female Voice in Comic Book Narrative. Lanham: Scarecrow Press, Inc.

[ref92] Denzin N.K. (2009). The Research Act: A Theoretical Introduction to Sociological Methods. New Brunswick & London: Aldine Transaction.

[ref17] Duncan-Jones K. (1998). ‘O happy dagger’: the autonomy of Shakespeare’s Juliet. Notes Queries 45, 314–316. doi: 10.1093/nq/45.3.314

[ref18] Elkins J. (ed.). (2009). Visual Literacy. New York & Oxon: Routledge.

[ref88] Espinosa R. (2008). Graphic Shakespeare Romeo and Juliet. Adapted from Shakespeare by Joeming Dunn. Cover page. Minnesota: Magic Wagon. Available at: https://www.amazon.co.uk/Romeo-Juliet-Graphic-Shakespeare-William/dp/1602701938 (Accessed November 30, 2022).

[ref19] Fardouly J. Diedrichs P. C. Vartanian L. R. Halliwell E. (2015). Social comparisons on social media: the impact of Facebook on young women’s body image concerns and mood. Body Image 13, 38–45. doi: 10.1016/j.bodyim.2014.12.002, PMID: 25615425

[ref130] First Global Workshop for the Voluntary National Reviews (2019). UN High-level Political Forum on Sustainable Development in 2020. UN: Oslo, Norway. 1–13.

[ref20] Fisher N. Frey D. (2007). Reading for Information in Elementary School: Content Literacy Strategies to Build Comprehension. Prentice-Hall. Five Astonishing Facts About Women in Shakespeare. Oxford University Press (OUPblog).

[ref21] Fleming J. (2016). “The ladies’ Shakespeare,” in A Feminist Companion to Shakespeare. ed. Callaghan D. (Wiley Blackwell: Sussex).

[ref22] Fokum V. Y. Fonjong L. N. Adams M. J. (2020). Increasing women’s representation in the Cameroon parliamentary: do numbers really matter? Women's Stud. Int. Forum 80:102369. doi: 10.1016/j.wsif.2020.102369

[ref23] Frossard J. (2012). A (graphic) novel approach to teaching Shakespeare: embracing non-traditional texts in the secondary English classroom. Student Works 91. Available at: https://scholarsarchive.byu.edu/studentpub/91?utm_source=scholarsarchive.byu.edu%2Fstudentpub%2F91&utm_medium=PDF&utm_campaign=PDFCoverPages

[ref89] Fuchs E. Otto M. Yu S. (2020). Textbooks and inclusive education. Paris: UNESCO.

[ref24] Fu Q. Hwang G. (2018). Trends in mobile technology-supported collaborative learning: a systematic review of journal publications from 2007 to 2016. Comput. Educ. 119, 129–143. doi: 10.1016/j.compedu.2018.01.004

[ref26] Gunakumaran S. (2003). “Literature programmes in Malaysian schools: a historical overview,” in The Teaching of Literature in ESL/EFL Contexts. eds. Ganakumaran S. Edwin M. (Petaling Jaya: Sasbadi Sdn. Bhd), 27–48.

[ref25] Ganakumaran S. Ismail S. Lee K. Y. (2003). “The incorporation of the literature component in Malaysian schools,” in The Teaching of Literature in ESL/EFL Contexts. eds. Ganakumaran S. Edwin M. (Petaling Jaya: Sasbadi Sdn. Bhd), 62–87.

[ref120] Goold, R. (Director) (2010). Macbeth. [DVD]. Illuminations Media and BBC.

[ref27] Hamilton S. (1943). Shakespeare’s Daughter. London: McFarland & Company.

[ref28] Hayley E. (2010). “Manga Shakespeare,” in Manga: An Anthology of Global and Cultural Perspectives. ed. Johnson-Woods T. (New York: Continuum).

[ref29] Hodgdon B. (1998). The Shakespeare Trade: Performances and Appropriations. Philadelphia: University of Pennsylvania Press.

[ref30] Inness S.A. (1999). Tough Girls, Women Warriors and Wonder Women in Popular Culture: Feminist Cultural Studies, the Media and Political Culture. Philadelphia: University of Pennsylvania Press.

[ref31] Isa N. H. Mahmud C. T. (2012). Literary texts for Malaysian secondary schools: needs versus policy. Int. J. Humanit. Soc. Sci. 2, 76–86.

[ref32] Ismail H. H. (2017). Tracing representations of deviant masculinity in selected EighteenthCentury English novels. PhD thesis. Universiti Putra Malaysia.

[ref33] Ismail H. H. (2019). Literature and gender equality as National Sustainable Goals. Trends Soc. Sci. 1, 16–24.

[ref34] Johnson-Woods T., (2010). Manga: An Anthology of Global and Cultural Perspectives. New York: Continuum.

[ref35] Kemp T. D. (2010). Women in Shakespeare’s World in Women in the Age of Shakespeare. Oxford: Greenwood Press.

[ref36] Khoo H. S. Knight P. (2015). Teachers’ evaluation of KBSM form 4, 5 English textbooks in review used in the secondary schools in Penang, Malaysia. Adv. Lang. Literary Stud. 6, 128–150.

[ref37] Kitsa M. Mudra I. (2020). Gender stereotypes of women in television advertising in Ukraine. Fem. Media Stud. 20, 381–397. doi: 10.1080/14680777.2019.1574857

[ref38] Kocher P. H. (1954). Lady Macbeth and the doctor. Shakespear. Q. 5, 341–349. doi: 10.2307/2866013

[ref39] Kumar J. (1989). Women in Indian television serials: issues of character, representation and acceptance. PostScriptum: an interdisciplinary. J. Literary Stud. 5, 37–47.

[ref40] Lichter C. (2007). Manners, intellect, and potential: a historiography on the underachievement of boys in literacy. Counterpoints 315, 3–15.

[ref41] Lim K. P. Lye C. T. Yuen Y. Y. Teoh W. M. Y. (2019). Women directors and performance: evidence from Malaysia. Equal. Divers. Inclus. 38, 841–856. doi: 10.1108/EDI-02-2019-0084

[ref128] Lynch, M. (2016a). The Three Witches Graphic Shakespeare Competition. [Image]. Facebook. Available at: https://www.facebook.com/photo/?fbid=10153543805164397&set=pcb.1052682694784446 (Accessed November 30, 2022).

[ref600] Lynch M. (2016b). The Three Witches Graphic Shakespeare Competition. [Image]. Facebook. Available at: https://www.facebook.com/photo/?fbid=10153543805274397&set=pcb.1052682694784446 (Accessed November 30, 2022).

[ref42] Matthison S. (1988). Why triangulate? Educ. Res. 17, 13–17. doi: 10.3102/0013189X017002013

[ref43] McCloud S. (1993). Understanding Comics: The Invincible Art. New York: Harper Perennial.

[ref44] McGarr S. P. T. (2008). Representations of Deficient Motherhood in English Novels of the Eighteenth Century: Daniel Defoe, Samuel Richardson, Frances Burney, and Ann Radcliffe. Unpublished Doctoral thesis. Griffith University.

[ref45] McKeon M. (1995). Historicizing patriarchy: the emergence of gender difference in England, 1660-1760. Eighteenth Cent. Stud. 29, 295–322.

[ref46] Mills J. S. Musto S. Williams L. Tiggemann (2018). Selfie harm: effects on mood and body image in young women. Body Image 27, 86–92. doi: 10.1016/j.bodyim.2018.08.007, PMID: 30149282

[ref47] Mohaideen M. S. Ismail H. H. Rashid R. A. (2020). The use of local literary texts as Reading materials in English language classrooms: an analysis of teachers’ perspectives. Int. J. Learn. Teach. Educ. Res. 19, 127–144. doi: 10.26803/ijlter.19.11.8

[ref48] Mortimore-Smith S. R. (2012). “Shakespeare gets graphic: reinventing Shakespeare through comics, graphic novels and manga,” in Locating Shakespeare in the Twenty-First Century. eds. Malcom G. Marshall K. (Newcastle: Cambridge Scholars).

[ref49] Mukundan J. (2007). Evaluation of English language textbooks: some important issues for consideration. J. NELTA 12, 80–85.

[ref50] Mukundan J. Haji Mohammadi R. Nimehchisalem V. (2011). Developing an English language textbook evaluation checklist. Contemp. Issues Educ. Res. 4, 21–28. doi: 10.19030/cier.v4i6.4383

[ref51] Mukundan J. Nimehchisalem V. (2008). Gender representation in Malaysian secondary school English language textbooks. Indones. J. English Lang. Teach. 4, 65–83.

[ref52] Murakami S. Bryce M. (2009). Manga as an educational medium. The international journal of the. Humanities 7, 47–56. doi: 10.18848/1447-9508/CGP/v07i10/42761

[ref53] Musa N. Husin A. Muhd Amin A. S. (2018). Semakan Dasar Wanita Kelantan: Penambahbaikan Pelaksanaan ke arah Mencapai Matlamat Pembangunan Mampan. Akademika 88, 137–150. doi: 10.17576/akad-2018-8803-12

[ref54] Nambiar R. M. K. Ibrahim N. Hashim R. S. Yasin R. M. Azman H. Yusof N. M. (2020). Impact of local culture-based Reading materials on students’ skill development and confidence in English. Univ. J. Educ. Res. 8, 445–453. doi: 10.13189/ujer.2020.080215

[ref55] Nayar P. K. (2017). Editor’s introduction: gender and the graphic. Indian J. Gend. Stud. 24, 231–235. doi: 10.1177/0971521517697882

[ref56] Okubo K. Sato K. Wada Y. Asai K. Kubo S. Horita T. (2019). Study of manga Reading as an effective teaching method based on the text comprehension process. Int. J. Learn. Technol. Learn. Environ. 2, 54–66. doi: 10.52731/ijltle.v2.i2.383

[ref87] O’Hagan C. (2021). When schools shut: Gendered impact on COVID-19 school closures. Paris: UNESCO.

[ref57] Öz H. Efecioğlu E. (2015). Graphic novels: an alternative approach to teach English as a foreign language. J. Lang. Linguist. Stud. 11, 75–90.

[ref81] Parkinson B. Thomas H.R. (2000). Teaching Literature in a Second Language. Edinburgh: Edinburgh University Press. doi:10.1515/9781474471534

[ref58] Pingel F. (2009). UNESCO Guidebook on Textbook Research and Textbook Revision. 2nd *Edn*. George Eckert Institute for International Textbook Research: Braunschweig.

[ref123] Polanski, R. (Director) (1971). Macbeth. [motion picture]. Columbia Pictures.

[ref59] Pope D. J. Butler H. Qualter P. (2012). Emotional understanding and color-emotion associations in children aged 7-8 years. Child Dev. Res. 2012, 1–9. doi: 10.1155/2012/975670

[ref60] Ramet S. P. (1996). Gender Reversals and Gender Culture. New York: Routledge.

[ref61] Roth E.H. Aberson T. (2006). Compelling Conversations: Questions and Quotations on Timeless Topics. Charleston, USA: Chimayo.

[ref62] Sani A. M. Zain Z. (2011). Relating adolescents’ second language reading attitudes, self efficacy for reading, and reading ability in a non-supportive ESL setting. Read. Matrix 11, 243–254.

[ref63] Shanahan N. Brennan C. House A. (2019). Self-harm and social media: a thematic analysis of images posted on three social media sites. BMJ Open 9, 1–6. doi: 10.1136/bmjopen-2018-027006, PMID: 30782950 PMC6367987

[ref64] Sinha M. S. Malshe M. (2017). Graphic novels as pedagogical tools in the Indian classroom: teachers’ opinions. Lang. Lang. Teach. 6, 1–6.

[ref125] Sixth Malaysia Plan (1991). Kuala Lumpur: National Printing Department. Available at: https://www.epu.gov.my/en/economic-developments/development-plans/rmk/sixth-malaysia-plan-1990-1995 (Accessed October 25, 2022).

[ref90] Skierso A. (1991). Textbook selection and evaluation. In M. Celce-Murcia. *Teaching English as a second or foreign language* (pp. 432-453). (2nd Ed.). Boston: Heinle & Heinle Publishers.

[ref65] Specht H. (2015). The beautiful, the handsome and the ugly: some aspects of the art of character portrayal in medieval literature. Stud. Neophil. 56, 129–146. doi: 10.1080/00393278408587888

[ref126] Sustainable Development Goals Report (2021). MUnited Nations Statistics Division 2021. Available at: https://unstats.un.org/sdgs/report/2021/

[ref66] Status of Ratification (n.d.). Convention on the elimination of all forms of discrimination against women. United nation. Available at: https:://tbinternet.ohchr.org/_layouts/15/TreatyBodyExternal/Treaty.aspx?Treaty=CEDAW&Lang=en (Accessed October 11, 2022).

[ref67] Tamada K. (2008). A consideration of learning with the use of comics–from viewpoints of psychology with comics and psychology on comics. Manga Stud. 3, 65–75.

[ref68] Tomlinson B. (1993). Material Development in Language Teaching. Cambridge: Cambridge University Press.

[ref69] Tso A. W. (2016). Teaching Shakespeare to young ESL learners in Hong Kong. J. Pedag. Dev. 6, 18–24.

[ref70] UNESCO (1995). Declaration and Integrated Framework of Action on Education for Peace. Paris: Human Rights and Democracy.

[ref73] Unser-Schutz G. (2011). “Manga as a linguistic resource for learning,” in *JALT2010 Conference Proceedings*. ed. A. Stewart (Tokyo: JALT).

[ref74] Vethamani M. E. (2005). Towards a literary tradition: Malaysian literature in English. Nationhood Literat. Express. Real. 1, 1–19.

[ref85] Volley W. Devlin J. Campbell J. (2008). Macbeth: The Graphic Novel. Adapted from Shakespeare by John N. McDonald. Cover page. Ludlow: Classical Comics. Available at: https://www.amazon.com/Macbeth-Graphic-Novel-Original-Text-ebook/dp/B00CJ0B0JY (Accessed November 30, 2022).

[ref86] Volley W. Devlin J. Campbell J. (2009). Romeo and Juliet The Graphic Novel. Adapted from Shakespeare by John N. McDonald. Cover page. Ludlow: Classical Comics. Available at: https://www.amazon.com/Romeo-Juliet-Graphic-Novel-Quick-ebook/dp/B01DMJYPRY (Accessed November 30, 2022).

[ref124] Wright, G. (Director) (2006). Macbeth. [motion picture]. Mushroom pictures.

[ref75] Yeong P. J. (2018). How women matter: gender representation in Malaysia’s 14th general election. Round Table 107, 771–786. doi: 10.1080/00358533.2018.1545943

[ref76] Yoshida S. (2013). Manga as teaching material. Bull. Educ. Res. Setsunan Univ. 9, 25–34.

[ref77] Younger M. R. Warrington M. (2006). Would Harry and Hermione have done better in single-sex classes? A review of single-sex teaching in coeducational secondary schools in the United Kingdom. Am. Educ. Res. J. 43, 579–620. doi: 10.3102/00028312043004579

[ref78] Yusof M. S. Lazim Zalina M. Salehuddin K. (2017). Teacher trainees’ perspectives of teaching graphic novels to ESL primary schoolers. 3L 23, 81–96. doi: 10.17576/3L-2017-2303-06

[ref79] Zainuddin A. Khalid K. (2018). Konsep Mengarusperdanakan Gender: Kajian Wanita dalam Pekerjaan di Malaysia. J. Administrat. Sci. 15, 1–10.

[ref80] Zanettin F. (2010). “Humour in translated cartoons and comics,” in Translation, Humour and the Media. ed. Chiaro D. (London & New York: Continuum).

